# Chemical Diversity and Microbial Complexity of Liupao Tea Aroma: A Comprehensive Review

**DOI:** 10.3390/foods15162832

**Published:** 2026-08-14

**Authors:** Shengxue Ye, Chunhong Piao, Hai Huang, Jingwang Wu, Chengying Jiang, Yang Song

**Affiliations:** 1 College of Food Science and Pharmaceutical Engineering, Wuzhou University, Wuzhou 543003, China; 18486374577@163.com (S.Y.);; 2 College of Light Industry and Food Engineering, Guangxi University, Nanning 530004, China

**Keywords:** Liupao tea, volatile compounds, microbiome, key odor-active compounds, metabolic mechanism

## Abstract

Liupao tea (LPT), one of China’s representative fermented dark teas, develops its unique “red, heavy, aged, and mellow” flavor characteristics. This review provides a comprehensive update on LPT aroma, integrating analyses of volatile organic compounds (VOCs), associated microorganisms, odor-active compounds (OACs), and metabolic pathways of key odor-active compounds (KOACs). With over 2000 VOCs documented, predominated by alcohols (368), hydrocarbons (357), and ketones (351), the total for LPT substantially exceeds the approximately 1000 compounds typically reported for other dark teas. By combining sensory evaluation with techniques such as gas chromatography–olfactometry (GC-O) and odor activity value (OAV), KOACs, predominantly comprising alcohols, aldehydes, and methoxybenzene derivatives, were identified as key contributors to the floral, fruity, aged, stale and other notes that define LPT’s characteristic aroma. The formation of the aroma compounds is closely linked to microbial metabolism and the enzymatic activities generated by microorganisms. Fungi, including *Aspergillus*, *Rhodotorula*, *Geosmithia*, *Wallemia*, *Penicillium*, *Blastobotrys*, *Fusarium*, and *Xeromyces*, and bacteria, including *Burkholderia*, *Ralstonia*, and *Achromobacter*, serve as the primary functional microorganisms driving fermentation and aging. This review provides a comprehensive framework for understanding the chemical and microbial complexity of LPT aroma.

## 1. Introduction

**Figure 1:**
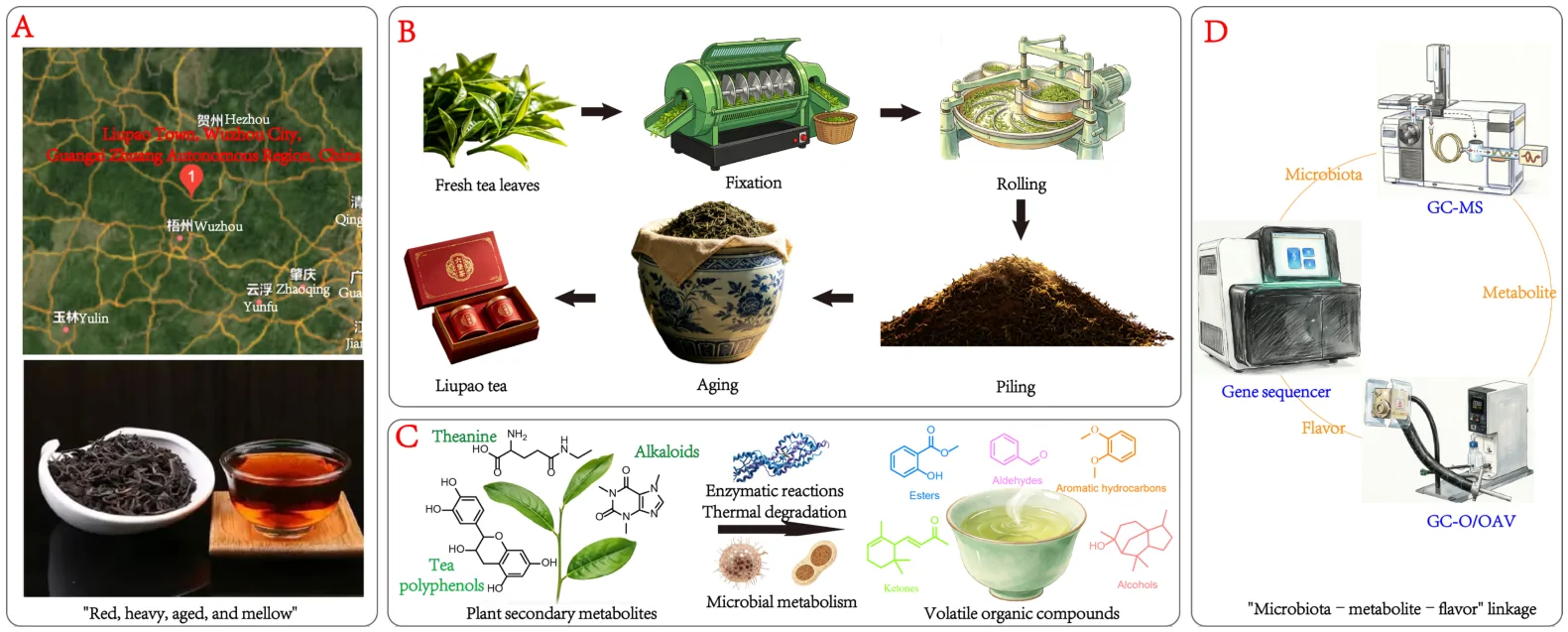
(**A**) The origin and characteristics of LPT. (**B**) Major manufacture processes of LPT. (**C**) Mechanisms of aroma formation in Liupao tea. (**D**) “Microbiota–metabolite–flavor” linkage of LPT.

Liupao tea (LPT), which originated in Liupao Town, Wuzhou City, Guangxi Zhuang Autonomous Region, China, is a typical microbial-fermented dark tea that has been consumed for over 1500 years [1]. LPT is widely recognized as the celebrated “overseas Chinese tea”, occupying an important role in overseas markets and embodying substantial cultural exchange value. LPT is now exported to over 40 countries and regions, with major markets including Malaysia, Singapore, Japan, the United States, and Europe. The comprehensive output value of the LPT industry has exceeded 25 billion RMB, providing employment for over 110,000 people. Its average export price has consistently remained above the national average for tea exports. In contrast to green tea and black tea, the distinctive quality of LPT is primarily developed during its unique pile-fermentation and long-term storage and aging process (Figure 1B) [2]. During these processes, the chemical components in tea undergo profound transformations under the synergistic action of microorganisms and enzymes, giving rise to the characteristic quality traits of LPT, namely “red, heavy, aged, and mellow” (Figure 1A).

Tea aroma is a key determinant of its quality and consumer preference, with its chemical essence being a complex mixture of hundreds of VOCs [3]. These compounds originate from plant secondary metabolites and can undergo profound changes during processing through pathways such as enzymatic reactions, thermal degradation, and microbial metabolism [4]. For LPT, the post-fermentation and aging processes are the essence of its flavor formation. During this prolonged process, the activity of endogenous tea enzymes gradually diminishes, while the metabolic activity of exogenous microorganisms becomes the dominant force driving the transformation of chemical components [2]. These microbial communities not only reshape the non-volatile matrix of tea (Figure 1C)—such as reducing catechin content and increasing theabrownins and alkaloids—but more importantly, they generate a lot of VOCs with distinctive aromas by decomposing macromolecular substances and transforming small-molecule precursors in tea leaves [5].

In recent years, techniques such as gas chromatography–mass spectrometry (GC-MS), solid-phase microextraction (SPME), and stir bar sorptive extraction (SBSE) have been widely used to identify VOCs in tea [6,7]. Combined with GC-O and OAV calculations, these methods enable the screening of key aroma-active compounds that truly contribute to the overall aroma from complex chemical profiles. Meanwhile, the application of high-throughput sequencing technologies allows for a comprehensive characterization of the microbial community structure and its dynamic succession patterns during different fermentation and aging stages of LPT [5]. By performing correlation analyses between volatile metabolomics and microbiome data, researchers are gradually establishing the “microbiota–metabolite–flavor” linkage (Figure 1D), providing strong evidence for elucidating the mechanisms of aroma formation [8]. Here, “microbiota” refers to the microbial community structure, “metabolite” denotes the volatile organic compounds (VOCs) profiled by analytical techniques, and “flavor” represents the sensory outcome driven by key odor-active compounds. Correlation analyses between microbiome and metabolome are typically performed using Pearson/Spearman correlation, OPLS-DA, and Mantel tests, with the aid of software such as R packages (vegan v2.6-4, corrplot v0.92, ggcor v0.9.9), SIMCA v14.1, and Cytoscape v3.10.1.

Although certain progress has been made in previous studies, a systematic understanding of the formation of the unique aroma of LPT remains insufficient. For LPT, elucidating the chemical composition of its VOCs, revealing their dynamic evolution patterns during processing and aging, and identifying the key compounds that confer its characteristic aroma are of critical importance for establishing a scientific quality evaluation system, optimizing production processes, and achieving targeted flavor regulation [2,4]. Therefore, this article synthesizes existing research findings and provides a systematic review and discussion on the formation of the unique aroma of LPT from three perspectives—the profiling of volatile aroma components, the role of microbial communities, and the formation mechanisms of key aroma compounds—aiming to provide reference and inspiration for future research in this field.

## 2. Volatile Compounds in LPT

**Figure 2:**
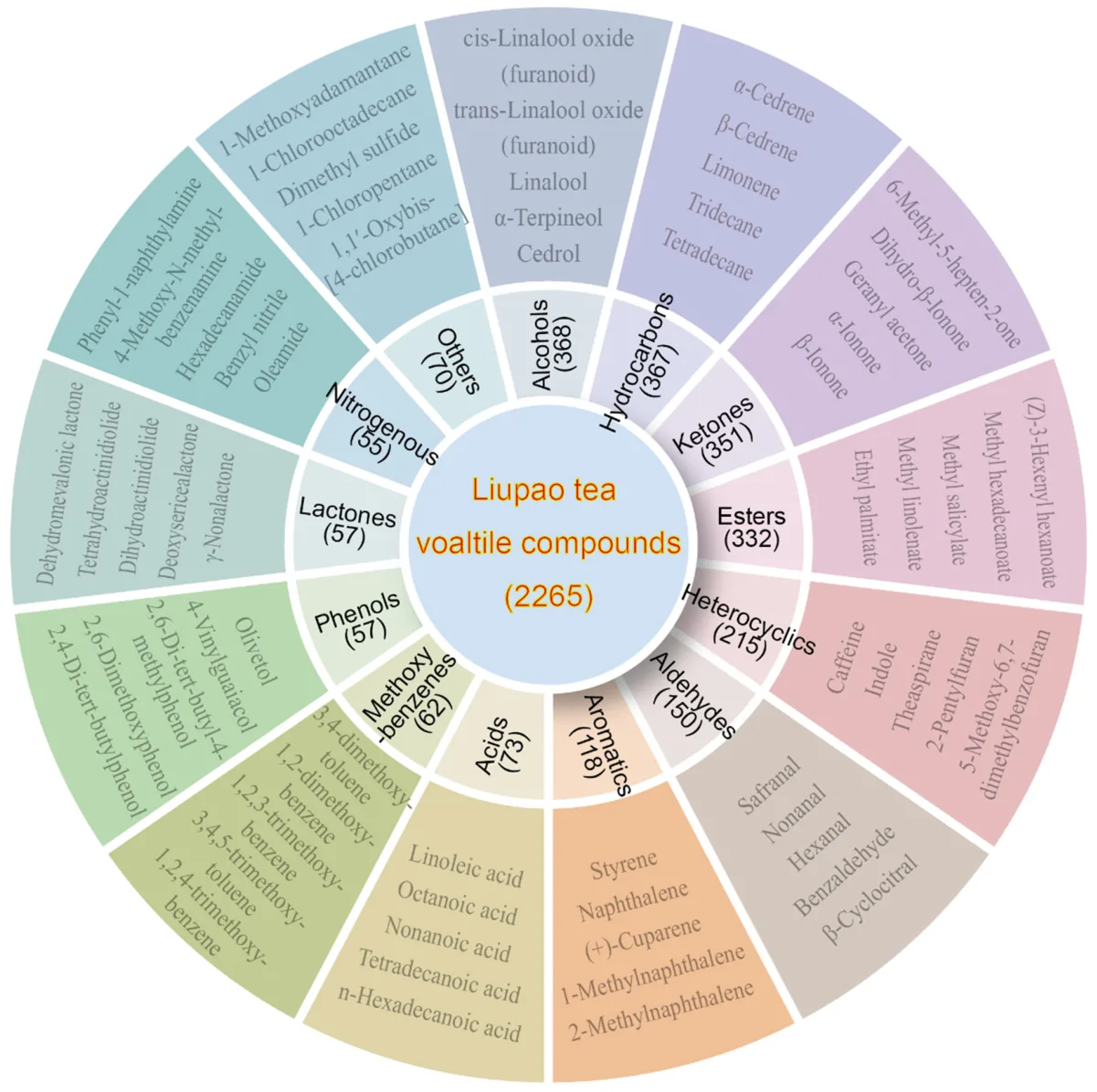
Volatile compounds have been documented in LPT. (The compounds shown in the figure are those with the highest detection frequencies within each chemical group).

Flavor is one of the core indicators for evaluating food quality, and VOCs constitute the material basis of food aroma [3]. These compounds are diverse and present at extremely low concentrations. However, slight changes in their composition and relative abundance can result in significant differences in the overall aroma characteristics [4]. In recent years, with the continuous advancement of analytical techniques, research on the VOCs in tea has become increasingly in-depth. Current literature has cataloged over 2000 VOCs in LPT, including alcohols (368), hydrocarbons (357), ketones (351), esters (332), heterocyclic compounds (215), aldehydes (150), aromatic hydrocarbons (118), acids (73), methoxybenzene compounds (62), phenols (57), lactones (57), nitrogenous (55), and others (70) (Table S1 and Figure 2).

To compile the volatile compound dataset for this review, a systematic literature search was conducted in the Web of Science, PubMed, and China National Knowledge Infrastructure (CNKI) databases, covering publications up to June 2026. A total of 44 primary studies were retrieved and included for data extraction. Peer-reviewed original research articles that applied GC-MS, SPME, SBSE, or related analytical techniques to identify VOCs in LPT were included. Reviews without primary data, studies lacking detailed compound identification, and reports not published in English or Chinese were excluded. The VOCs reported in these 44 studies were compiled and summarized to generate the dataset. For each compound, the detection frequency (DF) was calculated as the number of studies in which it was reported. A DF threshold of ≥5 was applied to select the most consistently detected compounds, thereby minimizing incidental reports and focusing on compounds with robust documentation across independent investigations.

It should be noted that the aging conditions of LPT, including temperature, relative humidity, and ventilation, are not standardized and may vary considerably among different production regions, storage facilities, and aging strategies. These environmental factors can significantly influence the rate and extent of chemical transformations during aging [1], and consequently affect the resulting volatile composition and sensory characteristics. In the studies cited below, the specific aging conditions are generally consistent within each study, but variations across studies may account for some differences in reported compound dynamics.

### 2.1. Alcohols

Alcohol aroma compounds can not only make the aroma of tea rich and persistent, but they also give the tea infusion a mellow and sweet aftertaste [9]. Alcohol compounds that contribute predominantly floral, fruity, and woody notes to the volatile profile are important components of the volatile aroma compounds in LPT [10]. Alcohols, with 368 compounds identified to date, are the most abundant volatiles in LPT. Among these, the most frequently detected (DF ≥ 5) are linalool (42), α-terpineol (40), cedrol (37), cis-linalool oxide (furanoid) (34), trans-linalool oxide (furanoid) (24), geraniol (23), 1-octen-3-ol (23), 2-phenylethanol (21), terpinen-4-ol (20), benzyl alcohol (16), phytol (15), 2-ethylhexanol (14), trans-linalool oxide (pyranoid) (14), nerol (13), β-cyclohomogeraniol (12), α-cadinol (11), (E)-nerolidol (11), hotrienol (9), cis-linalool oxide (pyranoid) (8), nerolidol (8), linalool oxide (8), isophytol (7), endo-borneol (7), 1-hexanol (7), 1-octanol (7), 2,6-dimethylcyclohexanol (6), 1-hexadecanol (6), 2-heptanol (6), geosmin (6), epicedrol (5), p-cymen-8-ol (5), fenchol (5) and 1-pentanol (5). The numbers in parentheses are the DF.

The presence of linalool and its oxides has been associated with enhanced floral characteristics and suppressed roasted odors in tea. Linalool and its oxide level increase during storage [11]. Among them, cis-Linalool oxide (furanoid) occurs at levels roughly 37% higher in LPT than in other dark teas [12,13], and this compound is considered a key driver of LPT’s characteristic betel nut note. α-Terpineol with a contributor to a fruity note, observed to occur broadly in vegetables and fruits [14], was also regarded as an important aroma compound in LPT [15]. However, its content decreases throughout the aging period [6,16]. Terpinen-4-ol exhibits clove-like odors and has been implicated in the formation of characteristic aroma in fermented tea [17]. Cedrol, a key flavor compound with a cedarwood odor (floral, dry, sweet, and soft) in both Fu brick and LPTs [18,19,20], plays a crucial role in developing the characteristic “stale” aroma [16]. Interestingly, studies have shown that its content increases significantly during the aging of Fu brick tea [21], yet it exhibits a decreasing trend of LPT [6], implying that this divergence likely stems from inherent differences between the two types of tea in terms of raw materials, processing regimens, and aging practices [22]. Geraniol is identified as an important flowery aroma compound in LPT and green tea [23]. Microbial activity during aging may sustain the hydrolysis of glycosidic precursors, thereby driving a progressive rise in geraniol levels [24]. 1-Octen-3-ol, which imparts mushroom and earthy odors and also found in blooming black tea, is a characteristic aroma compound of LPT [25]. 2-Phenylethanol exhibits a pleasant rose-like floral aroma [26]. Benzyl alcohol accounts for a substantial portion of the aromatic compounds found in tea [27]. Phytol (floral, powdery) content decreases during microbial fermentation of LPT [28]. 2-Ethylhexanol has been singled out as the principal odorant imparting chestnut-like notes [29]. Likewise, nerol characterized by its minty scent has been reported as a significant contributor to the flavor profile of citrus tea [30]. α-Cadinol exhibits woody odors and makes major contributions to the woody attribute of LPT [31]. The content of (*E*)-nerolidol with floral aroma increases significantly after 15 h of piling [32]. Nerolidol exhibits floral, fruity, and sweet aroma [33]. Borneol, which imparts a minty note, is a common component in peppermint essential oil. Beyond its widespread application as a spice and natural preservative in foods and herbal teas, borneol also acts as a flavor enhancer [18]. 1-Hexanol levels rise progressively with prolonged storage [34] and the specific odors have not been reported. In contrast, geosmin with an exceptionally high OAV of 5551.28 is considered the primary driver of the earthy note in LPT [6]. Odor activity value (OAV) is defined as the ratio of the concentration of a compound to its odor detection threshold. Typically, compounds with OAV > 1 are considered odor-active, while those with OAV ≥ 10 are regarded as having high odor activity and are likely to make substantial contributions to the overall aroma profile.

### 2.2. Hydrocarbons

Hydrocarbons rank second in abundance among LPT volatiles, with 357 compounds documented to date. Those detected in at least five studies (DF ≥ 5) include α-cedrene (20), β-cedrene (15), limonene (15), tridecane (13), tetradecane (12), pentadecane (12), δ-cadinene (12), neophytadiene (10), hexadecane (10), heptadecane (9), farnesene (9), D-limonene (9), β-ocimene (9), dodecane (8), terpinolene (8), β-myrcene (8), cedrene (7), β-elemene (7), γ-terpinene (7), phytane (7), octadecane (6), caryophyllene (6), thujopsene (6), (E)-β-ocimene (6), α-selinene (5), β-cadinene (5), β-guaiene (5), decane (5), longifolene (5), β-funebrene (5), anthracene (5) and 3-ethyl-1,4-hexadiene (5).

Saturated hydrocarbon compounds (tetradecane, hexadecane, and so on) generally possess high odor thresholds and low odor intensities, thus contributing little to the overall aroma [35]. The content of α-cedrene with sweet aroma decreases significantly during the early stage of piling, which might be the main reason for the diminished sweet aroma in tea [32]. The unsaturated hydrocarbon β-cedrene possesses a woody aroma and is also present in relatively high content in LPT [10]. Cedrene, caryophyllene, and δ-cadinene all exhibit woody aroma and have been identified as contributors to the woody attribute of LPT. Caryophyllene contributes appreciably to the aromatic intricacy and richness of tea [36]. Farnesene arises from secondary metabolic biosynthesis and imparts a complex bouquet of citrus, herbal, and lavender notes [37]. D-limonene and limonene exhibit fresh orange aroma [38]. β-Elemene has been reported as a major contributor to the woody note of *Aphanamixis polystachya* essential oil [39]. γ-Terpinene with oily woody odor have been identified as the representative compounds of LPT [28]. For the other hydrocarbons, only trace levels are present in LPT. Their odor descriptions are accordingly unavailable, most likely due to their limited concentrations.

### 2.3. Ketones

Ketone compounds also play an important role in tea aroma, often exhibiting floral, fruity, or woody characteristics. As the third-largest group of VOCs, ketones account for 351 of the VOCs documented in LPT. Those with DF ≥ 5 encompass α-ionone (32), β-ionone (32), geranyl acetone (29), 6-methyl-5-hepten-2-one (27), dihydro-β-Ionone (23), 2,2,6-trimethylcyclohexanone (21), 6, 10, 14-trimethyl-2-pentadecanone (20), isophorone (19), (*E*, *E*)-3,5-octadien-2-one (16), acetophenone (16), 2-undecanone (15), dehydro-β-ionone (12), 2-heptanone (12), trans-β-ionone (11), jasmone (11), 5,6-epoxy-β-ionone (11), (+)-2-bornanone (10), 3,5-octadien-2-one (9), 6,10-dimethyl-5,9-undecadien-2-one (8), benzophenone (8), 2-acetylpyrrole (7), 6-ethenyldihydro-2,2,6-trimethyl-2H-pyran-3(4H)-one (7), 2,6,6-trimethyl-2-cyclohexene-1,4-dione (6), 6-methyl-3,5-heptadiene-2-one (5), farnesylacetone (5) and 1-ethyl-2,5-pyrrolidinedione (5).

The carotenoid derivatives—α-ionone, β-ionone, trans-β-ionone, dihydro-β-ionone, dehydro-β-ionone, and 5,6-epoxy-β-ionone—exhibit woody and floral odors and have been implicated as key drivers of characteristic aroma formation in various types of tea [40,41]. Among them, the flavor significance of β-ionone has been well established in LPT as well as in Pu-erh tea [42]. Trans-β-ionone and dihydro-β-ionone significantly diminish after aging [6], with trans-β-ionone having an extremely low odor threshold [5]. Geranyl acetone exhibits a fresh and refreshing rose-like aroma [43] and has been documented as a significant volatile constituent across LPT [44,45,46]. 6-Methyl-5-hepten-2-one and acetophenone always decrease during storage [36]. 6-Methyl-5-hepten-2-one, known for its floral and sweet notes, is an aroma-active constituent of LPT, oolong tea infusions, and aged Longjing tea [25,47]. 2,2,6-Trimethylcyclohexanone with floral–fruity aroma and 2-heptanone with blue cheese note show progressive accumulation during LPT aging [34]. Isophorone occurs at high abundance in post-fermented dark teas as a common metabolite of microbial fermentation [48], yet its concentration declines appreciably in the later stages of the process [49]. (*E*, *E*)-3,5-Octadien-2-one, which possesses sweet and vanilla-like notes, is primarily responsible for the sweet taste of LPT [50,51]. 2-Undecanone is characterized predominantly by floral, fruity, and woody notes [47]. Jasmone, which exhibits a jasmine odor, has been reported as a key aroma compound in LPT, Chin-Shin oolong tea and Four Seasons tea [46,52]. (+)-2-Bornanone has been frequently reported in long-stored LPT, where its camphoraceous note is thought to enhance its herbal character [18,53]. 6,10-Dimethyl-5,9-undecadien-2-one exhibits floral and fruity aromas [10]. 2-Acetylpyrrole is thought to originate from arginine and proline metabolism, and its accumulation may diminish the floral and fruity notes of tea [54].

### 2.4. Esters

Esters are the primary contributors to fruity and floral aromas. Esters, with 332 compounds identified to date, rank as the fourth-most abundant volatile class in LPT. The subset with DF ≥ 5 encompasses methyl salicylate, methyl hexadecanoate, methyl linolenate, ethyl palmitate, (Z)-3-hexenyl hexanoate, diisobutyl phthalate, methyl linoleate, diethyl phthalate, dibutyl phthalate, vinyl hexanoate, cis-3-hexenyl benzoate, methyl stearate, 2,2,4-trimethyl-1,3-pentanediol diisobutyrate, methyl benzoate, benzyl benzoate, methyl anthranilate and benzyl acetate.

Certain ester compounds, such as methyl hexadecanoate, methyl linoleate, and methyl linolenate, are formed by the dehydration condensation of higher fatty acids and lower alcohols. These compounds exhibit low volatility and are odorless, thereby contributing little to the aroma of tea [55]. Methyl salicylate, which exhibits wintergreen and mint-like odors, is not only present in LPT, but has also been detected in other types of dark tea [22]. The content of methyl salicylate increases significantly after 15 h of piling [32]. (*Z*)-3-hexenyl hexanoate (fruity green pear) is generally much higher in dark raw tea than in various dark teas [22]. Methyl benzoate exhibits a clove oil-like aroma. Together with methyl salicylate, it belongs to the class of aromatic esters, and both serve as aroma sources of betel nut note LPT [56]. These substances greatly enrich the aromatic complexity of LPT, facilitating its transition from a simple green and floral aroma to a complex aged and fruity note.

### 2.5. Heterocyclic Compounds

Heterocyclic compounds are typically formed during thermal processing or microbial fermentation and exert significant effects on the flavor of food products. Among the heterocyclic compounds detected in LPT, those with a detection frequency of 5 or more include 2-pentylfuran, theaspirane, caffeine, indole, 5-methoxy-6,7-dimethylbenzofuran, dibenzofuran and caryophyllene oxide.

Furan compounds contribute relatively little to the overall aroma formation of post-fermented tea [57]. 2-Pentylfuran, characterized by a typical green note, is recognized as a contributor to both the stale and betel nut aroma profiles of LPT [18]. Theaspirane with green and fresh has been identified as the representative compounds of LPT [28]. Caffeine is related to the taste of tea, which makes little contribution to the aroma of tea [55]. Indole, which possesses an orange- and jasmine-like floral aroma, is present in relatively high concentrations in Tieguanyin, Huangjingui, and Dahongpao [55]. Caryophyllene oxide with woody notes have attracted attention as potential broad-spectrum pharmacological agents [58]. Heterocyclic compounds contribute a mellow, sweet taste and aroma to the tea infusion.

### 2.6. Aldehydes

Aldehydes typically have low odor detection thresholds, which makes them particularly impactful contributors to the overall aroma. A total of 150 aldehydes have been cataloged in LPT, of which those with DF ≥ 5 include benzaldehyde, safranal, β-cyclocitral, nonanal, hexanal, decanal, benzeneacetaldehyde, (*E*, *E*)-2,4-heptadienal, heptanal, *(E*)-2-octenal, (*E*)-2-hexenal, (*E*, *E*)-2,4-decadienal, (*E*)-2-nonenal, octanal, 1-ethyl-1H-pyrrole-2-carboxaldehyde, β-apo-8-carotenal, 2-undecenal, (*E*, *Z*)-2,6-nonadienal and (*E*)-2-decenal.

Benzaldehyde and benzeneacetaldehyde, both characterized by bitter almond notes [59], are associated with aging and are considered indicators of microbial activity that enriches the complexity of the aged aroma. Benzaldehyde, a key contributor to fruity notes, accumulates during LPT storage, thereby intensifying this sensory attribute [60,61]. Benzeneacetaldehyde, which possesses fruity and floral notes, is regarded as one of the most reliable odorants responsible for the chestnut-like aroma of green tea [51]. This compound is thought to derive from phenylalanine oxidation [62], and its concentration has been proposed as a potential criterion for tea quality grading [63]. Safranal and 1-ethyl-1H-pyrrole-2-carbaldehyde exhibit green odors [19]. β-Cyclocitral possesses herbal, sweet, and tobacco-like odors [64]. Nonanal, associated with aged and fungal aromas, exhibits elevated levels in LPT [65,66]. Hexanal with grassy, caramel, and peppermint characteristics had lower odor activity values [49], being important for forming tea aromas [67]. Decanal, which exhibits herbal and fatty odors, have been detected as a contributor to the overall aroma formation of post-fermented tea [42], and the levels significantly increase during the late stage of LPT fermentation [49]. (*E*, *E*)-2,4-Heptadienal always decreases during storage [36]. Heptanal, which contributes a green note, is commonly detected in dark tea [60,68]. (*E*)-2-Octenal and (*E*)-2-decenal attribute such as fruity, grassy, and herbal notes [47]. (*E*)-2-Hexenal exhibits green and fruity notes in LPT [33]. (*E*, *E*)-2,4-Decadienal has been shown to generate new aromatic constituents upon baking of LPT, likely through the oxidative breakdown of linoleic and linolenic acid lipids in the tea leaves [38]. (*E*)-2-Nonenal exhibits fatty and green-like odors and has been detected with a high dilution factor in Pu-erh tea [9]. Octanal, which imparts a pronounced fruity note, contributes substantially to the fruity character of LPT [69]. The relative content of 1-ethyl-1H-pyrrole-2-carboxaldehyde in the raw material stage is as high as 11.87% [10]. (*E*, *Z*)-2,6-nonadienal is recognized for enhancing the cucumber and green notes of tea infusion and is regarded as a key odorant in both Chinese black tea and dark tea [49].

### 2.7. Aromatic Hydrocarbons

Aromatic hydrocarbons, with 118 compounds identified to date, represent a minor VOC class in LPT. The DF ≥ 5 subset includes naphthalene, 1-methylnaphthalene, 2-methylnaphthalene, styrene, (+)-cuparene, 1,1,6-trimethyl-1,2-dihydronaphthalene, toluene, α-curcumene, fluorene, 3,4-diethyl-1,1′-biphenyl, 1,6-dimethylnaphthalene, o-xylene, m-xylene, p-xylene, 2,2′,5,5′-Tetramethylbiphenyl, ethylbenzene, 1,7-dimethylnaphthalene, o-cymene, p-cymene, cadalene and α-calacorene.

Naphthalene contributes pungent, dry, and tarry notes to dark tea. However, its relatively high odor threshold and low odor activity mean that its overall sensory impact remains negligible [49]. 1-Methylnaphthalene exhibits pungent note [18]. 2-Methylnaphthalene exhibits smoky or burnt notes, yet carries a more pronounced green character than naphthalene [18]. Toluene (plastic-like) and fluorene (naphthalene-like) both decline markedly in concentration as LPT ages [6]. 1,1,6-Trimethyl-1,2-dihydronaphthalene possesses aged and woody aroma characteristics [15]. O-cymene exhibits floral note. These compounds may contribute significantly to the unique aroma of LPT, warranting future investigation in this direction.

### 2.8. Methoxybenzene Compounds

Methoxybenzene compounds play a dual role in tea aroma: they mitigate the coarse and aged notes of parched green tea while also contributing to the characteristic stale and musty profile of LPT and Pu-erh tea [26,70,71]. Furthermore, the aroma characteristics of stale-aroma type of LPT was similar with that of Pu-erh tea [18]. Methoxybenzene compounds, with 62 species identified to date, constitute a minor but sensorially significant VOC class in LPT. The DF ≥ 5 subset includes 1,2-dimethoxybenzene, 1,2,3-trimethoxybenzene, 3,4-dimethoxytoluene, 3,4,5-trimethoxytoluene, 1,2,4-trimethoxybenzene, 4-ethyl-1,2-dimethoxybenzene, 1,2,3,4-tetramethoxybenzene, 1,3-dimethoxybenzene, methyl eugenol, anethole, elemicin, estragole, 3,4-dimethoxystyrene, methyl isoeugenol and 3,5-dimethoxytoluene.

In relation to dark teas, compounds such as 1,2-dimethoxybenzene 1,2,3-trimethoxybenzene, 3,4-dimethoxytoluene, 1,2,4-trimethoxybenzene, 4-ethyl-1,2-dimethoxybenzene, 1,2,3,4-tetramethoxybenzene, and 1,3-Dimethoxybenzene, which carries a stale note, has been reported as a key aroma compound in earlier investigations [46]. Among these compounds, 1,2-dimethoxybenzene appears to reinforce the stale character indirectly through its herbal note [6]. In contrast, 3,4-dimethoxytoluene acts in a concentration-dependent manner: it amplifies both stale and earthy notes at low levels, but its impact on staleness becomes more dominant at higher concentrations. Furthermore, 3,4,5-trimethoxytoluene (burnt plastic, spice) declines markedly in concentration as LPT ages, and this reduction appears to attenuate the smoky note [6]. As the aging time progresses, the contents of these methoxybenzene compounds gradually accumulate, rendering the aged aroma characteristic increasingly pronounced. The compound’s sensory influence thus shifts with concentration, affecting different attributes to varying degrees [6].

### 2.9. Acids, Phenols, Lactones, Nitrogenous and Others

Acids, phenols, lactones, nitrogenous, and other compounds are generally thought to play a relatively minor role in the overall aroma of LPT. To date, 73 acids, 57 phenols, 57 lactones, 55 nitrogenous compounds, and 70 other compounds have been reported in LPT. Among these, acids with a DF factor ≥5 include n-hexadecanoic acid, nonanoic acid, tetradecanoic acid and linoleic acid. Phenols with a DF ≥ 5 are olivetol, 2,4-di-tert-butylphenol, 2,6-di-tert-butyl-4-methylphenol and 2,6-dimethoxyphenol. Lactones with a DF ≥ 5 include dihydroactinidiolide and tetrahydroactinidiolide. The DF of nitrogenous and other compounds are all below 5.

Although the DF of n-hexadecanoic acid is 12, its contribution to the aroma of LPT has been rarely reported in the literature. In the study by [72], n-hexadecanoic acid has been identified as the most predominant organic acid compound in LPT and is also considered one of the most abundant aroma components in dark tea [73]. Nonanoic acid exhibits a faint fatty and coconut-like aroma, but its relative content is low, and no reports have addressed its contribution to the aroma of LPT [56]. 2,4-Di-tert-butylphenol, which imparts a characteristic phenolic odor, has been considered an active compound in LPT and other dark teas [43,49]. 2,6-Di-tert-butyl-4-methylphenol is primarily used as a food antioxidant. Its specific sources and contributions require further investigation [55]. Dihydroactinidiolide is widely distributed in dark tea and black tea [18]. Its content keeps stable [60] or shows an increasing trend [6] and it contributes a unique roast, flowery and fruity aroma during the LPT storage. Other volatiles, such as 1-p-menthene-8-thiol and benzenemethanethiol, are notable for their sulfurous and onion-like odors—sensory attributes rarely encountered in other tea beverages. Despite their very low concentrations, these compounds stand out prominently in the volatile profile of finished LPT [49].

The remaining VOCs with DF ≥ 5 have been detected in LPT but lack documented odor descriptions. Together with other components (Table S1), they add to the overall aromatic complexity [74,75,76,77,78,79,80,81,82,83,84,85,86,87,88,89,90,91].

## 3. Formation Mechanism of Key Odor-Active Compounds in LPT

**Figure 3:**
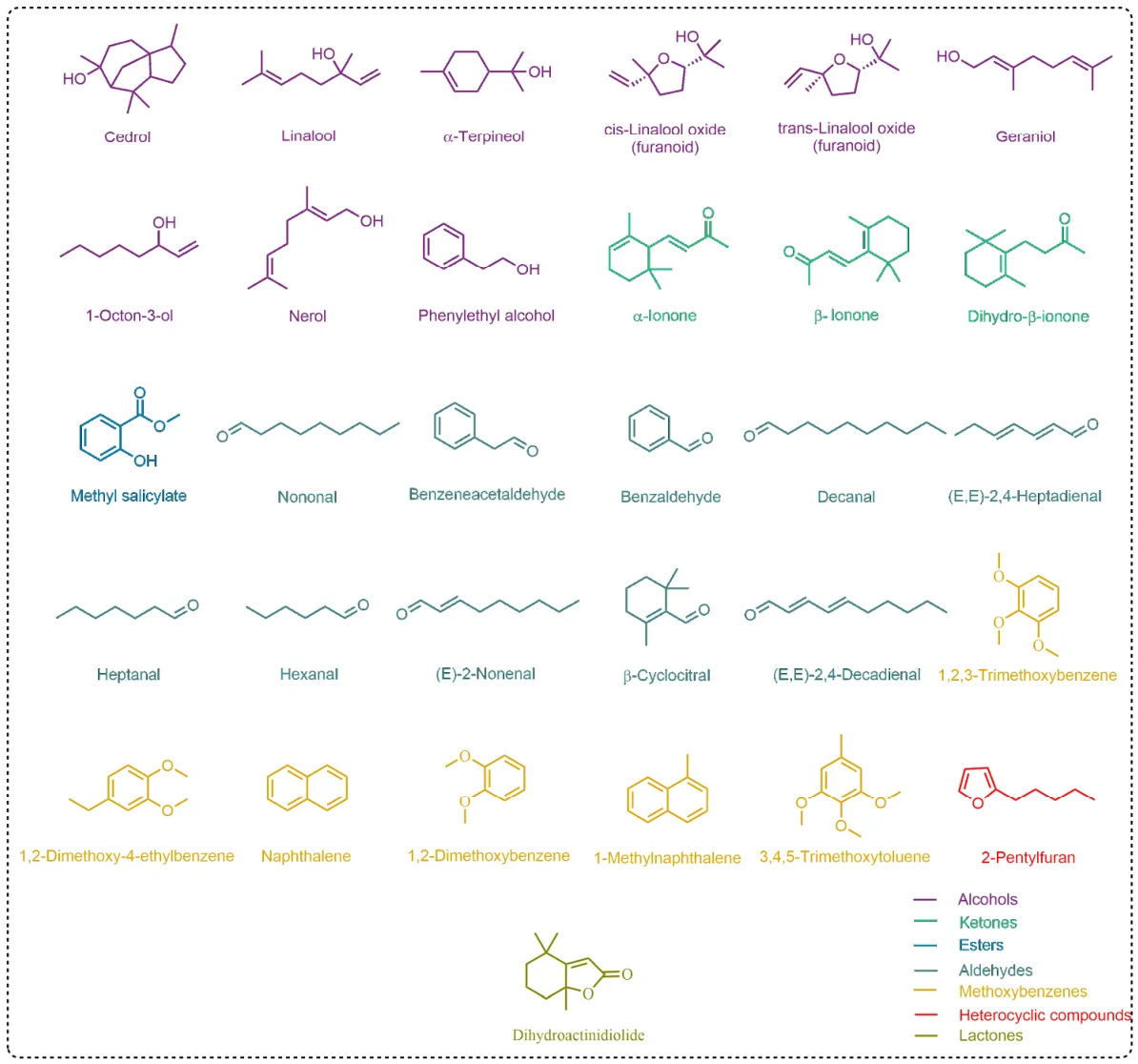
The chemical structure of key odor-active compounds.

The identification of key odor-active compounds (Figure 3) currently relies primarily on GC-O olfactometry and OAV calculations. The critical link between the microbiome and the aroma profile lies in the metabolic activities of the microorganisms. Fungi and bacteria produce a vast arsenal of extracellular enzymes that break down complex, non-volatile substrates in the tea leaves into smaller, volatile molecules. Elucidating these metabolic pathways is key to understanding and controlling aroma formation. The key odor-active compounds with a DF ≥ 5 are shown in Figure 3 and Table S2.

### 3.1. Alcohols

**Figure 4:**
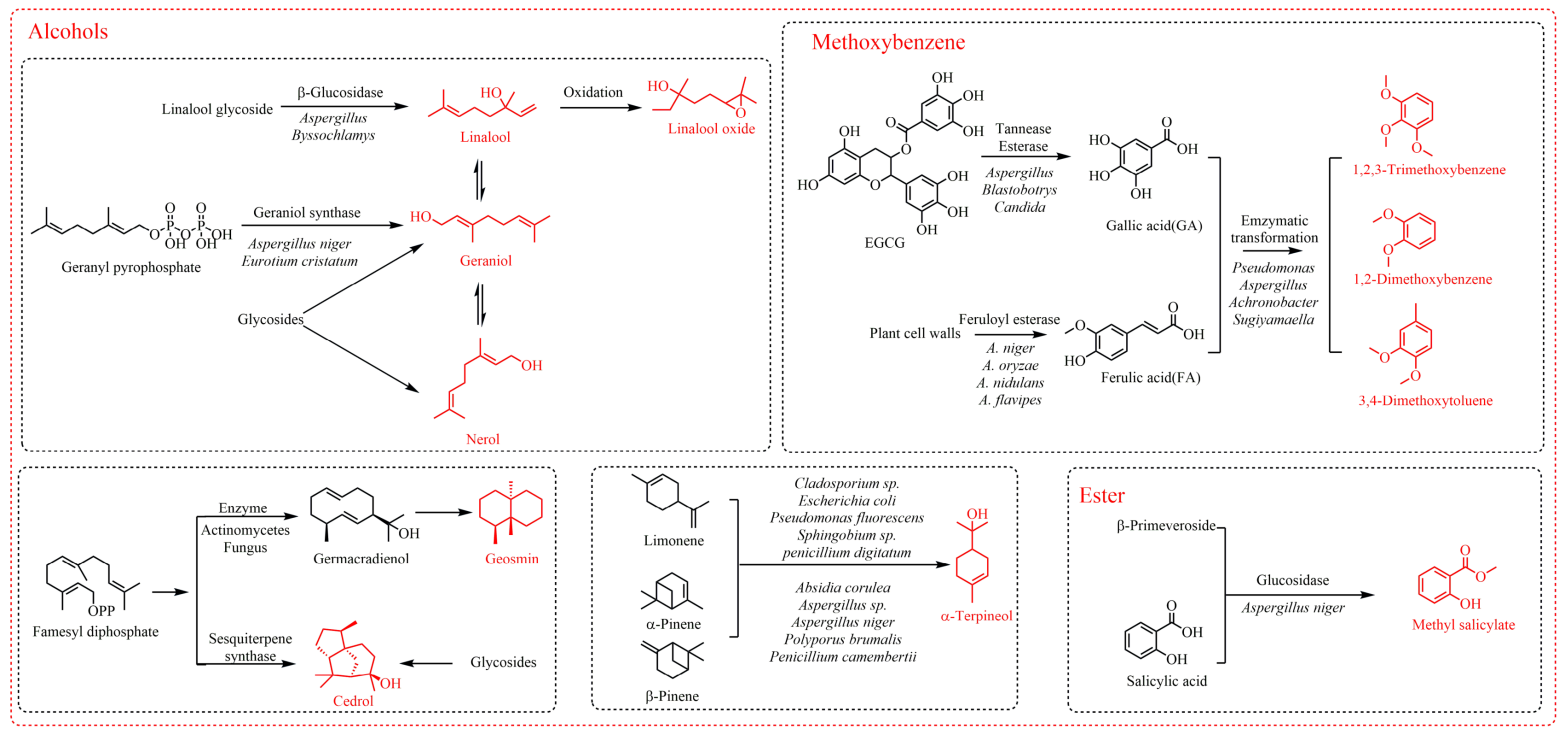
Possible formation pathways of key odor-active compounds (Aocohos, methoxybenzenes, and esters).

Due to the inherent volatility, most alcohol compounds, which possess floral and sweet aromas, predominantly showed a decreasing trend with increasing storage time [36]. However, linalool concentrations showed a slight increase with prolonged storage. It was discovered that β-glucosidase, an enzyme produced by *Aspergillus* and *Byssochlamys*, is critically involved in hydrolyzing cyclolinalool glycosides and phenylethyl alcohol glycosides. Interestingly, β-glucosidase exhibits a higher hydrolytic affinity toward β-glucopyranosides than toward phenylethyl alcohol glycosides [54]. Thus, while most alcohols including phenylethyl alcohol decline steadily during storage, linalool exhibits a slight accumulation (Figure 4). Linalool oxides, primarily present as β-primeveroside conjugates, originate from β-glucopyranosides via glucosidase-mediated hydrolysis and linalool oxidation [92]. Geosmin is a metabolic byproduct of cyanobacteria, fungi, and actinomycetes [93]. In microbial biotransformation, α-terpineol can be derived from the bicyclic monoterpenes α-pinene, β-pinene, and limonene [94]. Sales et al. [95] reported that *Absidia corulea*, *A. niger*, *P. camemberti*, and *Polyporus brumalis* participate in the biotransformation of α- and β-pinenes.

### 3.2. Ketones

**Figure 5:**
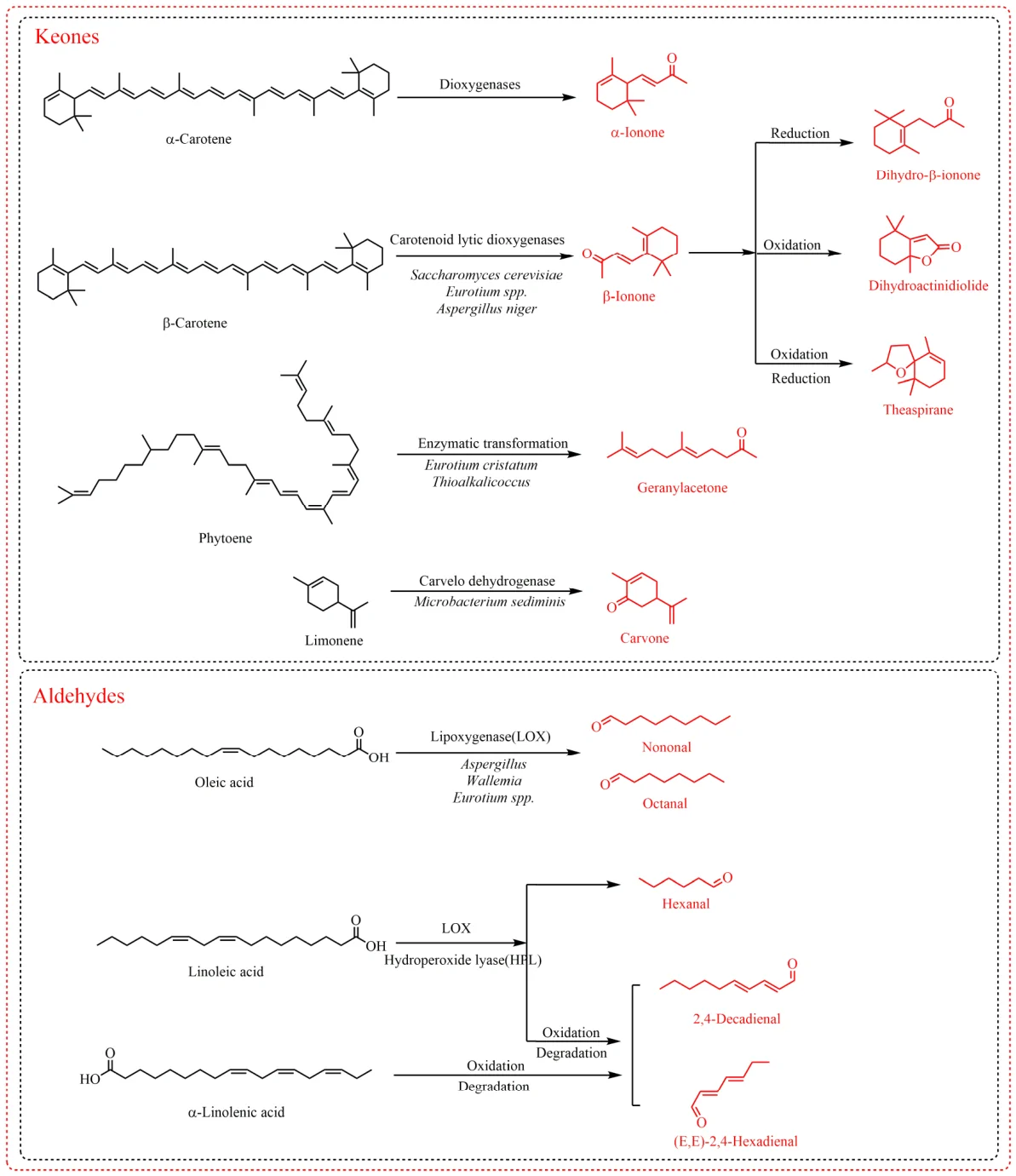
Possible formation pathways of key odor-active compounds (Keones and aldehydes).

Ketones in LPT are mainly carotenoid-derived volatiles (CDVs), generated from precursors including β-carotene, α-carotene, phytoene, lutein, and lycopene via the action of carotenoid cleavage dioxygenases (CCDs) during fermentation and drying—through enzymatic oxidation, photooxidation, autoxidation, or thermal degradation [96]. In LPT, α-Ionone derives mainly from α-carotene oxidative degradation and β-Ionone can arise from β-carotene via multiple routes: enzymatic reactions during pile fermentation, photooxidation, autoxidation during storage, and thermal degradation during fixing, drying, and steaming [97] (Figure 5). Dihydro-β-ionone and dihydroactinidiolide are derived from β-carotene as secondary oxidation products and from β-ionone through biotransformation. Carvone is produced from limonene via carveol dehydrogenase (EC 1.1.1.275), which showed a noticeable increase during pile fermentation [92].

### 3.3. Aldehydes

The primary sources of aldehydes are Strecker degradation and the breakdown of unsaturated fatty acids [43]. Some short-chain C6–C9 fatty aldehydes can be generated from fatty acid derivatives (FADVs) through the lipoxygenase (LOX) and hydroperoxide lyase (HPL) pathways [69,98]. Nonanal and octanal, both classified as FADVs, impart floral and fruity notes and are derived from the oxidative degradation of oleic acid. Hexanal probably came from the oxidation of linolenic acid [96]. Benzaldehyde typically originates from the hydrolysis of aroma glycosides, whereas benzeneacetaldehyde is thought to arise from the Maillard reaction [28]. (*E*, *E*)-2,4-heptadienal, (*E*, *Z*)-2,6-nonadienal, and (*E*, *E*)-2,4-decadienal arise from fatty acid breakdown in tea and contribute fatty and floral notes [46]. 2,4-Decadienal and (*E*, *E*)-2,4-heptadienal are formed as secondary degradation products from hydroperoxides derived from the oxidation of linoleic and α-linolenic acid during baking [38].

### 3.4. Others

Methoxybenzene compounds are thought to arise from the methylation of phenolic acid derivatives, catalyzed by microbial extracellular enzymes. Epigallocatechin gallate (EGCG) is converted to gallic acid (GA) by tannase and esterase, and GA is subsequently transformed into methoxybenzene compounds via microbes including *Aspergillus*, *Blastobotrys*, and *Candida* [99]. A reduction in GA levels is thought to enhance the release of methoxybenzene compounds and thereby contribute to the stale-aroma characteristic of LPT [6,100]. Ferulic acid (FA), the most abundant hydroxycinnamic acid in plant cell walls, may serve as an additional precursor for methoxybenzene compounds [94].

## 4. Role of Microbial Communities in LPT

The understanding of the LPT microbiome has undergone a transition from traditional culture-based methods to modern molecular biology techniques. Early research primarily relied on isolation and culture techniques, successfully identifying several dominant strains [101]. However, as most microorganisms are difficult to culture under laboratory conditions, these methods fail to fully capture the true microbial diversity. In recent years, the application of high-throughput sequencing technologies based on 16S rRNA and ITS gene amplicon sequencing, along with meta-omics approaches such as metagenomics and metatranscriptomics, has greatly deepened our understanding of the complexity of the LPT microbiome [102].

### 4.1. Mycobiome

Fungi play a dominant role during the fermentation process of LPT, serving as the primary agents that produce degradative enzyme systems and transform the internal components of tea leaves. Fungal genera with relatively high abundance in the LPT have been identified in previous studies, including *Aspergillus*, *Rhodotorula*, *Geosmithia*, *Wallemia*, *Penicillium*, *Blastobotrys*, *Fusarium*, *Xeromyces*, and unclassified fungus [5,18,103,104]. *Aspergillus* and *Wallemia* showed a high correlation with nonanal (r = –0.89 for *Aspergillus*, r = 0.83 for *Wallemia*), while *Aspergillus*, *Rhodotorula*, *Penicillium*, *Blastobotrys*, and *Fusarium* exhibited a strong correlation with linalool oxides, with correlation coefficients of −0.84, 0.97, 0.96, 0.83, and 0.84, indicating a close relationship with the formation of the betel nut aroma [103]. According to Ma et al., isophorone in LPT is related to core functional microorganisms such as *Aspergillus*, *Wallemia*, *Xeromyces* and *Blastobotrys* [28].

The sexual stage of *Aspergillus* (*Eurotium*) has been reported to contribute to the unique flavor formation of LPT [19]. A recent study found that *Aspergillus cristatellus* (*Eurotium cristatum*) is closely associated with the production of VOCs—including α-terpineol, furanoid trans-linalool oxide, and D-limonene—which possess floral, fruity, woody, and minty notes during tea leaf fermentation. *Blastobotrys adeninivorans* significantly influences the biotransformation of tea leaves and is capable of producing several heat-stable extracellular enzymes, including proteases, and glucoamylase that contribute to aroma development during LPT fermentation [18]. *Wallemia sebi* and *Wallemia muriae* have been closely linked to the flavor characteristics of fermented products and are also prevalent in light-flavor Daqu [105]. *Penicillium citrinum*, commonly detected in LPT, is used to produce several industrial enzymes—including those for enriching 5′-nucleotides in yeast extract flavorings, as well as cellulase and inulin-type fructooligosaccharides [106]. *Xeromyces bisporus*, the only known species within its genus, is regarded as a characteristic member of traditional fermentation starters and may help stabilize the aromatic profile of rice wine toward the end of fermentation [107].

Researchers have isolated and identified specific functional strains from LPT. For instance, *Aspergillus luchuensis* YZ-1, a strain isolated from LPT, has been demonstrated to possess promising probiotic potential, including tolerance to the gastrointestinal environment, adhesion ability to intestinal epithelial cells, as well as antimicrobial and antioxidant activities [108]. Overall, the fungal community in LPT fermentation exerts a dual role, driving aroma formation through enzymatic activities while also posing potential safety risks that necessitate proper process control, highlighting the importance of managing fermentation conditions to maximize benefits and minimize hazards.

### 4.2. Bacteriome

Bacteria also play an indispensable role in the fermentation ecosystem of LPT, acting synergistically with fungi to collectively influence tea quality. Dominant bacterial phyla include *Proteobacteria*, *Firmicutes*, *Bacteroidetes*, and *Actinobacteria* throughout the fermentation period. At the genus level, common taxa include *Bacillus*, *Lactobacillus*, *and Pantoea* [104]. For instance, polyphenol oxidase-producing strains have been isolated from the fermentation process of LPT, among which *Pantoea vagans* exhibited the highest enzyme activity [101].

*Burkholderia*, *Ralstonia*, *Achromobacter*, and unclassified Pseudonocardiaceae have been identified as core functional bacteria [60]. *Burkholderia*, an endophytic genus, is closely linked to polyphenol formation [109] and produces protocatechuate 3,4-dioxygenase, which participates in protocatechuate metabolism. Most Pseudonocardiaceae are thermophilic aerobes [110] capable of surviving in long-term, high-temperature piles of LPT. Many unclassified Pseudonocardiaceae secrete extracellular enzymes involved in the degradation of polyphenols and aromatic hydrocarbons [111]. Therefore, certain unclassified Pseudonocardiaceae may contribute to polyphenol degradation during prolonged LPT storage [60]. Consequently, the proportion of alcohols declined progressively during long-term storage, whereas that of aldehydes and ketones increased over the same period [60].

### 4.3. Microbial Interactions

**Figure 6:**
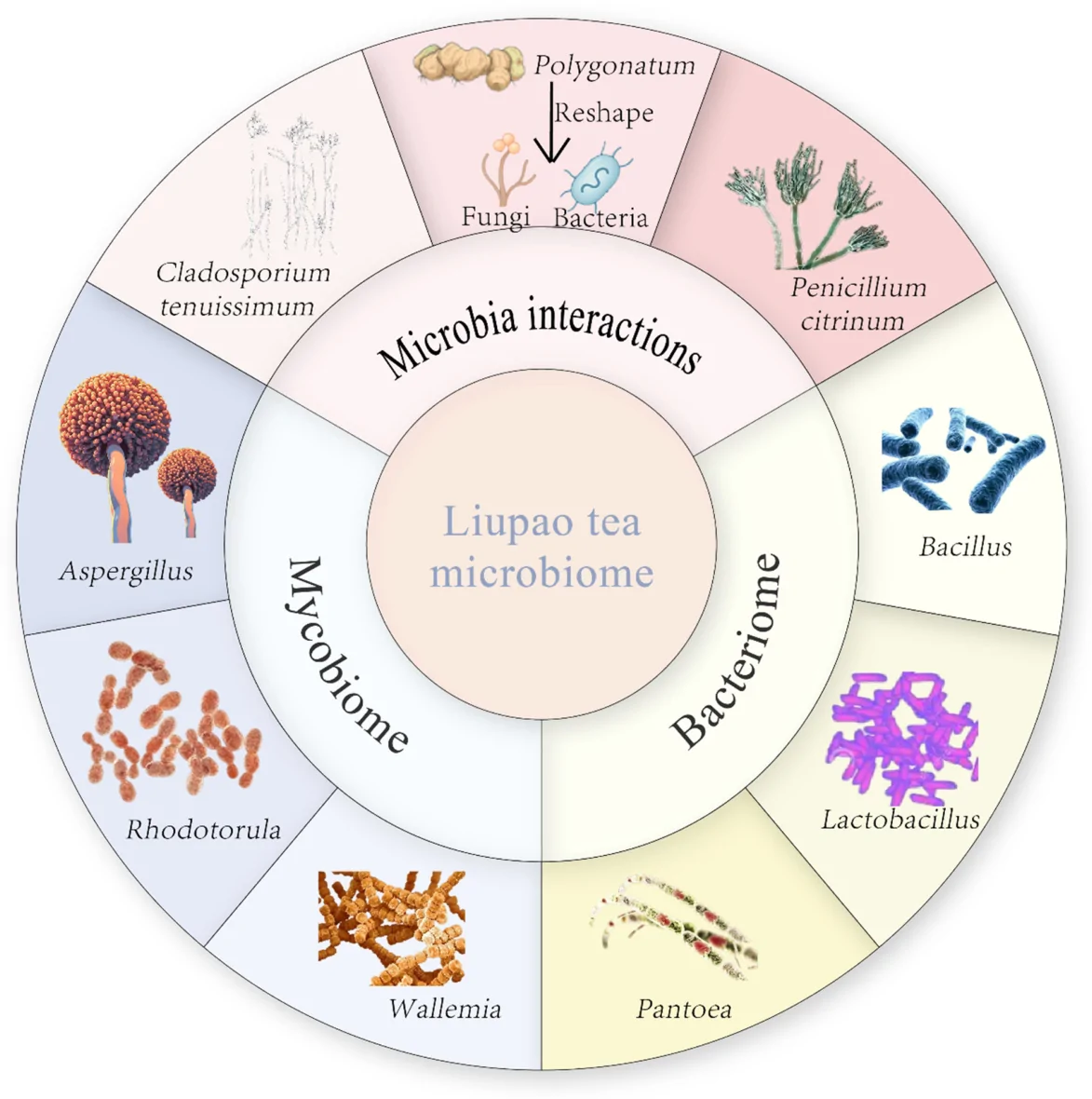
Major microorganisms involved in LPT fermentation.

External interventions can reshape the microbial community and pile environment of LPT. The inoculation with *Cladosporium tenuissimum* accelerated the aging of LPT, raised the content of (*E*, *E*)-3,5-octadien-2-one and decreased the levels of cedrol and 1,2,3-trimethoxybenzene, thereby shifting the aroma profile from stale to sweet-grassy [50]. The saccharified *Polygonatum* (an edible and medicinal plant) in LPT first stimulates saccharolytic, acid-tolerant bacteria hastening acidification and niche filtering, then the resulting warm, acidic pile favors thermotolerant fungi such as *Aspergillus* and *Thermomyces*, shifting the timing of their enrichment. Therefore, alcohol signals declined earlier while aldehydes rose sooner [112] (Figure 6).

These two intervention examples offer valuable insights into future targeted fermentation control strategies. The *Cladosporium tenuissimum* inoculation demonstrates the feasibility of bioaugmentation with specific fungal strains to accelerate aging and steer the aroma profile toward a desired direction, suggesting that systematic screening of functional microbes and directed inoculation could serve as a powerful approach for flavor-oriented fermentation management. The *Polygonatum* saccharification example illustrates that substrate modulation, through the addition of exogenous carbon sources, can reshape microbial community succession and metabolic dynamics, thereby adjusting the timing and intensity of volatile compound production. These complementary approaches highlight two promising avenues: bioaugmentation with functional microbes and substrate engineering to steer community function. Future research should focus on optimizing inoculation timing, dosage, and environmental parameters to achieve reproducible and predictable flavor outcomes.

The fermentation of LPT is a complex microbial ecological process involving both synergistic and competitive interactions between fungi and bacteria. As illustrated in Figure 6, the fungal community—dominated by genera such as *Aspergillus*, *Wallemia*, *Penicillium*, and *Blastobotrys*—works in concert with bacterial taxa including *Burkholderia*, *Ralstonia*, and unclassified Pseudonocardiaceae to drive the biotransformation of tea leaf components. These microorganisms secrete a wide array of extracellular enzymes, such as proteases, glucoamylase, and polyphenol oxidases, which break down macromolecular substrates into smaller volatile and non-volatile metabolites [4]. This enzymatic network forms the biochemical basis for the generation of key aroma compounds that collectively define the characteristic flavor profile of LPT.

Notably, the interactions between fungi and bacteria are not merely additive but often synergistic or competitive. For instance, certain bacteria may create favorable conditions for fungal growth by modulating local pH or producing specific metabolites that stimulate fungal activity. Conversely, small molecules such as monosaccharides generated by fungal decomposition of macromolecular substances can serve as nutrient sources for bacteria. This intricate interaction network collectively determines the direction of fermentation and the quality of the final product, a principle analogous to the mutualistic relationships observed in plant microbiomes [113]. A deep understanding of these interactive mechanisms is key to achieving precise regulation of the LPT fermentation process in the future.

## 5. Conclusions

The review synthesizes previous studies and paints a clear picture of LPT aroma as a product of a dynamic and complex microbial ecosystem. The unique sensory profile is not simply a result of the tea cultivar or initial processing but is actively constructed by fungi and bacteria during post-fermentation and aging. This “microbiome–aroma nexus” is a central tenet for understanding and controlling the quality of the post-fermented foods and beverages. To date, over 2000 VOCs have been documented in LPT, with alcohols (368), hydrocarbons (357), and ketones (351) being the most abundant categories, followed by esters (332), heterocyclic compounds (215), aldehydes (150), and aromatic hydrocarbons (118). While the numbers of acids (73), methoxybenzene compounds (62), phenols (57), lactones (57), nitrogenous (55), and others compounds are relatively small. Fungi including *Aspergillus*, *Rhodotorula*, *Geosmithia*, *Wallemia*, *Penicillium*, *Blastobotrys*, *Fusarium*, *Xeromyces*, and bacteria including *Burkholderia*, *Ralstonia*, *Achromobacter* and so on synergistically play an important role in aroma formation. Key odor-active compounds with DF ≥ 5 are cedrol, linalool, terpineol, cis-linalool oxide (furanoid), trans-linalool oxide (furanoid), geraniol, 1-octen-3-ol, nerol, phenylethyl alcohol, α-Ionone, β-ionone, dihydro-β-Ionone, methyl salicylate, nonanal, benzeneacetaldehyde, benzaldehyde, decanal, (*E*, *E*)-2,4-heptadienal, heptanal, hexanal, β-cyclocitral, (*E*)-2-nonenal, (*E*, *E*)-2,4-decadienal, 1,2,3-trimethoxybenzene, 1,2-dimethoxy-4-ethylbenzene, naphthalene, 1,2-dimethoxybenzene, 1-methylnaphthalene, 1,2,3-trimethoxy-5 methylbenzene, 2-pentylfuran, and dihydroactinidiolide. The formation mechanisms of LPT volatiles remain inadequately understood, and their precise biosynthetic routes have yet to be elucidated.

To date, over 2000 VOCs have been detected in LPT, collectively contributing to its unique aroma profile. This number is considerably higher than the 1000-plus compounds previously identified in Pu-erh tea [43] and Fu brick tea [114]. The exceptionally high diversity of VOCs documented in LPT not only highlights the chemical complexity of this tea category, but also underscores the challenges in establishing quality standardization. Meanwhile, the perceptual interactions of aromas in LPT also constitute an important factor influencing its overall flavor. Current studies predominantly rely on correlation-based approaches to link microbial taxa with VOCs. However, correlational evidence does not establish causation. To date, few investigations have employed functional genomic tools to experimentally validate the direct contribution of specific microbial taxa to LPT aroma formation. In addition, the concentration of 1,2-dimethoxybenzene and gallic acid followed a parallel trend during storage, suggesting a potential synergistic relationship between them in shaping LPT aroma [36]. Future research should integrate sensory evaluation with chemometric approaches to systematically elucidate the synergistic or antagonistic effects among key aroma compounds and to reveal the intrinsic link between microbial metabolism and perceptual interactions, thereby providing a theoretical basis for the targeted regulation of LPT flavor.

## Data Availability

No new data were created or analyzed in this study. Data sharing is not applicable to this article.

## Supplementary Materials

The following supporting information can be downloaded at: https://www.mdpi.com/article/10.3390/foods15162832/s1, Table S1: Volatile compounds identified in Liupao tea; Table S2: Odor-active compounds identified in Liupao tea.
